# Guilty Molecules, Guilty Minds? The Conflicting Roles of the Innate Immune Response to Traumatic Brain Injury

**DOI:** 10.1155/2012/356494

**Published:** 2012-06-04

**Authors:** Sarah Claire Hellewell, Maria Cristina Morganti-Kossmann

**Affiliations:** ^1^National Trauma Research Institute, The Alfred Hospital, 89 Commercial Road, Melbourne, VIC 3004, Australia; ^2^Department of Medicine, Monash University, Melbourne, VIC 3004, Australia; ^3^Department of Surgery, Monash University, Melbourne, VIC 3004, Australia

## Abstract

Traumatic brain injury (TBI) is a complex disease in the most complex organ of the body, whose victims endure lifelong debilitating physical, emotional, and psychosocial consequences. Despite advances in clinical care, there is no effective neuroprotective therapy for TBI, with almost every compound showing promise experimentally having disappointing results in the clinic. The complex and highly interrelated innate immune responses govern both the beneficial and deleterious molecular consequences of TBI and are present as an attractive therapeutic target. This paper discusses the positive, negative, and often conflicting roles of the innate immune response to TBI in both an experimental and clinical settings and highlights recent advances in the search for therapeutic candidates for the treatment of TBI.

## 1. Introduction

Traumatic brain injury (TBI) is a leading cause of death and disability, particularly in young adults who fall victim to motor vehicle accidents, falls, sporting injuries, and increasingly common assaults. Despite advances in prehospital and clinical care, a vast majority of severe TBI survivors will not be able to live independently or return to work [1]. Aside from the enormous personal burden of TBI, a substantial economic cost exists, estimated at $8.6 billion dollars each year in Australia alone [2] whilst in the United States this cost exceeds $55 billion dollars per year [3].

TBI has been described as the most complex disease in the most complex organ of the body; a sentiment which highlights both the multifactorial nature of brain injury in terms of type and spatial distribution of damage, and the intricacies of the brain's responses to insult. The pathology caused by a TBI can be classified in two broad temporal phases: the primary or initial injury to the head, which cannot be treated or prevented; the secondary injury, which is instigated by the primary injury, results in a complex cascade of pathophysiological and neurochemical events [4–6]. This ongoing secondary injury process is potentially amenable to intervention and, thus, has been the focus of research in the past two decades, with a view to halting or limiting these factors to avoid the progression of initial injury.

 Alas, many compounds showing promise in experimental models have shown largely disappointing results in the clinical setting [7, 8], and to date, no effective therapies exist to treat TBI [4]. This failure is likely due to the aforementioned complexities of the brain, and the propensity for use of rodents in preclinical trials of compounds, which overlooks the fundamental differences between human and rodent brains. Another key aspect has been the use of pharmacological agents that target a single factor of the complex interconnected pathways leading to secondary brain damage [9].

The immune system consists of two important components: the “innate” system, which is responsible for immediate, nonspecific action against pathogens or insults, and the “adaptive” system, a response tailored to the specific threat or insult at hand [10]. It is increasingly clear that far from being distinct, these systems are highly interrelated, with the innate system shaping and modifying the responses of the adaptive system [11]. Recently, the role of the innate immune system has been under the spotlight, as these early inflammatory responses implicitly designed to minimise the deleterious outcomes of injury have a somewhat paradoxical role in that they are increasingly implicated in the mediation of secondary pathogenic cascades.

The central nervous system (CNS) was traditionally thought to be a site of immune privilege due to the impermeable shield of the blood brain barrier (BBB). However, over the past two decades, it has been well established that under injury and inflammatory conditions, immune cells are able to cross the BBB and enter the brain parenchyma. The brain is also equipped with its own resident immune cells, the microglia, which undergo marked recruitment, proliferation, and activation in response to virtually any neuropathological insult [12].

This paper aims to provide an insight into the innate immune responses elicited by TBI, and the beneficial or detrimental roles these pivotal responses may exert in the pathogenesis of brain injury. We will also discuss therapies and strategies currently under investigation to minimise the inflammatory response to TBI or modulate it to a more beneficial phenotype.

## 2. Pathophysiological Responses to Traumatic Brain Injury

Initial or primary brain injury results in mechanical damage to the brain as a result of motor vehicle accidents, falls, sporting injuries, and violence [13]. The complex pathology caused by the primary TBI is further complicated by the intrinsic nature of the damage involved: focal or diffuse [5, 14]. Patients with focal injuries often present with skull fractures and subdural, epidural, or intraparenchymal haematomas [15], with the damage that occurs being largely dependent on the site of impact to the head. In contrast, diffuse brain injury is characterised by widespread damage to the white matter as well as the vasculature caused by acceleration/deceleration forces to the head [16]. Diffuse injury leads to axonal perturbation and impaired axonal transport, with gradual axonal disconnection from the soma [17]. Whilst patients with focal injuries are readily diagnosed using conventional CT scans, diffuse injuries often show no overt pathology and thus can potentially be missed during early imaging-assisted diagnosis [1, 7]. In addition, focal and diffuse injuries often coexist, particularly in motor vehicle accidents, falls, and assaults [5].

Both focal and diffuse TBIs can cooccur with insults such as hypoxia, hypotension and ischemia, or cerebral hypoperfusion [18–20]. These insults are commonly reported, occurring in approximately one-third of severe TBI patients [21] and are known to exacerbate pathology, with prolonged cognitive deficits and poorer long-term outcome when compared to patients experiencing an isolated TBI [22–25]. Animal studies have further elucidated this observation, with posttraumatic insults such as hypoxia and hypotension found to worsen behavioural outcomes and heighten pathology in models of both focal and diffuse injury [26–33].

At the time of the primary TBI, mechanical damage to the brain results in the activation of a multitude of pathways, including (but not limited to) excitotoxicity and oxidative stress, influx of Ca^2+^ and Na^+^, and efflux of K^+^ [34–36]. Subsequently, disruption of cell membranes, mitochondrial disturbance leading to energy failure, and a lack of ATP availability hamper reparative mechanisms the brain may attempt [37, 38]. High intracellular Ca^2+^ levels also trigger the activation of Ca^2+^-dependent proteases including calpains, caspases, and phospholipases, resulting in damage to the axonal cytoskeleton [39, 40]. Secondary injury cascades triggered by these primary injurious events include breakdown of the blood brain barrier (BBB) and extravasation of vascular fluid into the parenchyma, ultimately culminating in vasogenic oedema [41–43]. Increased BBB permeability facilitates the infiltration of peripheral immune cells and activation of resident immune cells, which release chemokines and cytokines and thus perpetuate the inflammatory response in the injured brain, with the end result of cellular dysfunction and death [44–46].

## 3. The Blood Brain Barrier Allows Transient Passage of Immune Cells into the Injured Brain

**Figure 1:**
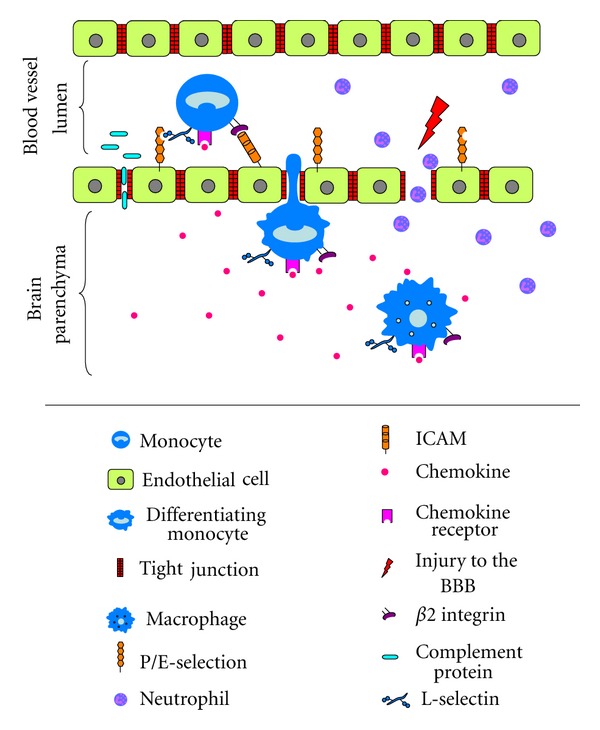
Passage of innate immune components through the blood brain barrier (BBB) after TBI. Injury to the brain results in transient opening of the BBB, in which complement proteins and neutrophils are able to directly enter the parenchyma. Peripheral monocytes enter the brain through a process of extravasation, in which several adhesion molecules are upregulated in turn on both the monocyte and endothelial cell to first tether, then provide passage for the cell through the BBB. First, constitutively expressed L-selectin binds to upregulated P/E-selectin on the endothelial cell surface. Once tethered to the endothelium, monocytes are exposed to chemokines that bind to their cognate receptors on the cell, inducing conformational change and upregulation of *β*2 integrins, which bind to ICAMs expressed on endothelial cells. This final interaction between adhesion molecules signals the cell to migrate across the endothelium into the parenchyma, where it begins to differentiate and take on the morphology of an activated macrophage. Under the influence of chemokines, the cell continues the transition to an activated macrophage state migrates to the site of injury. Figure adapted from [51].

The brain and the CNS have traditionally been considered to be sites of immunological privilege due to the BBB, however during certain inflammatory states, the BBB allows the transient passage of immune cells from the vasculature [47]. The BBB is composed of tight junctions at three sites: endothelial cells in the cerebral capillaries, the arachnoid barrier, and the blood-CSF barrier formed by the choroid plexus [48, 49] and is further defined by the associated cells—pericytes and astrocytes [49]. Under normal circumstances, the BBB tightly controls the exchange between plasma and the interstitial fluid, however the dysfunction caused by TBI allows for excess permeability, with disruption of tight junctions and transcytosis allowing passive diffusion. BBB disruption is typically transient, with an immediate period of hyperpermeability, in which immune cells and other products in the plasma may freely cross into the parenchyma [41, 50] (Figure 1).

## 4. Extravasation of Immune Cells into the Traumatically Injured Brain

Though peripheral immune cells may enter the CNS via the dysregulated BBB, the BBB is open for only limited periods of time, and thus cells must also cross the vasculature into the CNS via a process of extravasation. In focal TBI, neutrophils are the first immune cells to enter the injured brain, appearing first on the vascular endothelium within the first 24 hours of injury [52]. The passage of immune cells through the BBB to the parenchyma is mediated by adhesion molecules (Figure 1). These molecules, expressed on both the vascular endothelium and the immune cells themselves, are important mediators of brain injury as their expression and binding largely regulates the extent of peripheral immune cell entrance to the injured brain [53]. These adhesion molecules are sequentially upregulated to first tether, tightly adhere, and then provide passage for the cell through the vessel wall, beginning with P- and E-selectin expressed on the endothelium, whilst L-selectin is constitutively expressed on leucocytes [54]. Binding of these molecules tethers the cell to the endothelium, and, once secured, the cell is exposed to chemokines also present on the endothelium, which are highly upregulated in response to injury [55]. The binding of chemokines to their receptors on migratory cells induces conformational change and subsequent activation of the next family of adhesion molecules in the sequence, *β*2 integrins. These proteins, namely, CD11a (LFA-1), CD11b (Mac-1), and CD11c (p50.195) are expressed on the leucocyte cell surface and bind to endothelial cells expressing intercellular adhesion molecules (ICAMs) [53]. ICAMs belong to the immunoglobulin “superfamily” consisting of ICAM-1, ICAM-2, and vascular adhesion molecule (VCAM)-1, as well as ICAM-3, which is expressed on the leucocyte cell surface [54]. It is this binding which gives the final signal for extravasation of the cell through the endothelium into the parenchyma.

In rats, upregulation of E-selectin has been demonstrated on endothelial cells as early as 4 h after weight-drop injury and remained elevated until 48 h [56]. ICAM-1 has also been shown to be increased on the endothelium after weight-drop injury 4 h post-TBI [56]. In diffuse TBI, the number of ICAM-1 positive vessels was also increased by 4-fold compared to sham at 24 h [46]. This expression pattern showed late stage amplification, with an 8-fold maximal value observed at 4 days after injury, and only returning to sham levels 1 week after TBI [46].

Children suffering from TBI have also been found to have increased CSF levels of soluble ICAM-1, which correlated with poor outcome [57]. In our adult TBI study, we have reported that patients with large focal contusions had elevated levels of soluble ICAM-1 in their CSF, whilst interestingly, patients with small or absent lesions after TBI showed no such elevations [58]. These differences likely reflect the inconsistencies seen between distinct forms of TBI and may be indicative of the reported contrasts in inflammatory cell infiltrates in animal studies of focal and diffuse brain injuries, which will be discussed in more detail in the following sections. 

## 5. Innate Immune Cells in the Pathogenesis of Brain Injury

The innate cellular response to TBI involves both infiltrating and resident immune cells, which share many functions in resolving, and at times prolonging the pathological response to injury [11]. Each cell type involved is briefly discussed below.

### 5.1. Infiltrating Immune Cells

Neutrophils (often referred to as polymorphonuclear cells or leukocytes) are bone-marrow-derived cells which function to phagocytose cellular debris and bacteria [59]. They produce a number of factors designed to be harmful to bacteria and other pathogens, however these substances also have neurotoxic effects on mammalian cells and their release overtly contributes to tissue damage [47]. These molecules include reactive oxygen/nitrogen species (ROS/RNS), matrix metalloproteinases, and proinflammatory cytokines that perpetuate damage in the CNS [37]. After focal TBI, neutrophils are the first immune cell to cross the BBB and enter to sites of injury, though this response is short-lived, with a peak at 24–48 h after injury and a resolution in neutrophil numbers by 7 days [60–62]. Interestingly, diffuse TBI causes no such infiltration of neutrophils, with only sham-level numbers observed after injury in both immature and adult rats [46, 63].

*Monocytes/macrophages *are also bone marrow derived and contribute to neuroprotection and recovery after CNS injury by phagocytosing cellular debris and preserving healthy tissue. These cells have an important function in antigen presentation to T cells, and as such are also essential for activation of the adaptive immune response. Acutely after injury, infiltrating macrophages are able to produce growth factors and neurotrophins such as brain-derived neurotrophic factor (BDNF), nerve growth factor (NGF), insulin-like growth factor 1 (IGF1), and anti-inflammatory cytokines such as interleukin (IL)-10 and transforming growth factor-*β* (TGF *β*) [64]. However, these cells may also be neurotoxic to the injured brain, mediating glutamate release, generating ROS/RNS, and producing chemokines such as CXCL2, CXCL1, CXCL3, and CXCL8 to induce migration of neutrophils [65], and CCL-2 and RANTES to induce migration of monocytes [66]. Activated monocytes/macrophages are also key producers of proinflammatory cytokines such as tumor necrosis factor (TNF), IL-1*β*, and IL-6 [67]. As with neutrophils, the recruitment of monocytes is variable between focal and diffuse injury types, with substantially less monocyte recruitment after diffuse injury [52, 68].

### 5.2. The CNS Resident Innate Immune Cells

*Microglia *are the dynamic surveillance cells of the immune system, constantly exploring their environment for noxious agents and injurious processes [69, 70]. Microglia play a predominant role in the phagocytosis of cellular debris and respond to extracellular signals by functional transformation from a “resting” to an “activated” phenotype, in which their processes retract, making these cells morphologically and functionally indistinguishable from macrophages [71, 72]. Activated microglia are highly motile and able to rapidly move through the brain to sites of injury [70]. Several neuroinflammatory factors are able to stimulate microglial migration, including the chemokines CCL-2 and fractalkine [73–75] and complement anaphylatoxin C5a [76]. Microglia have long been scrutinized for their role in neuronal damage and particularly in synaptic stripping after TBI [77], however it has now been suggested that, rather than being the perpetrators of neuronal and axonal injury, it is more a case of “guilt by association,” since microglia may not be active participants in neuronal damage (for excellent review see [78]). This hypothesis has been corroborated by in vitro experiments of rat neuronal and microglial coculture, in which even when exposed to inflammatory factors, microglia did not cause direct neuronal damage [74].

*Astrocytes *are the most numerous cell type in the brain and become rapidly activated in response to injury in a process of “reactive astrogliosis,” in which cells undergo hypertrophy and proliferation proportional to injury severity [79, 80]. The role of astrocytes after TBI is controversial, as they are known to produce many proinflammatory cytokines including TNF, IL-1, and IL-6 and are also major producers of chemokines [81]. Astrocytes have also been shown to inhibit axonal spouting in lesioned tissue by formation of a dense fibrous glial scar [80, 82]. However, this glial scar also restricts tissue damage by forming a protective barrier, confining injury to a defined space and preventing further spread of damage [79, 83]. After TBI, astrocytes decrease the expression of glutamate transporters, with reduced glutamate uptake thus intensifying the excitotoxic response [84]. Conversely, reactive astrocytes upregulate the expression of matrix metalloproteinase (MMP) after TBI, and in particular release of MMP-3, which has been shown to be released from these cells in the vicinity of neurons undergoing synaptogenesis [85], suggesting that astrocytes may play a role in the clearance of damaged tissue in order to make a more permissive environment for neuronal plasticity.

## 6. Complement Proteins Are Pivotal in the Pathogenesis of Traumatic Brain Injury

Best known for its role in the recognition and elimination of pathogens, the complement system has recently emerged as a key innate mediator of the inflammatory response after brain injury. Complement is a complex network of soluble and cell-associated factors [48] and can be activated through three different pathways depending on the stimulus: the classical pathway, the alternative pathway, and the lectin pathway [86]. Within the CNS, complement has been shown to be upregulated both clinically in TBI patients and in various models of experimental TBI [87]. Under normal physiological conditions, complement proteins are detected at very low levels in the brain due to the precise compartmentalization of the vasculature and the parenchyma by the BBB [49], and thus peripheral complement proteins are unlikely to enter the brain without disruption of the BBB. After TBI, the disruption of the BBB allows an influx of serum complement proteins into the injured CNS [48, 87] (Figure 1). However, complement proteins can also be produced endogenously in the brain by astrocytes, microglia, and neurons in response to infection or injury [48].

Whilst the role of complement is intrinsically one of elimination and resolution of infection, the infiltration and/or activation of complement proteins after TBI may lead to inflammatory-induced damage by way of C3b deposition and subsequent opsonisation and phagocytosis, and C5a-induced recruitment and activation of immune cells from the periphery, with neutrophils being the “early responders” [88]. Overt tissue destruction may also occur with the final formation of the membrane attack complex (MAC), the primary role of which is mechanoporation [86]. Clinically, elevated levels of two crucial components of the alternative pathway, C3 and factor B have been demonstrated in the CSF of severe TBI patients, with concomitant BBB dysfunction in more than 50% of patients, suggesting that the elevated levels of C3 and factor B, were due to serum leakage across the dysfunctional BBB rather than *de novo* synthesis [89]. Similarly, C5b-9 (MAC), the cytolytic end product of the complement system has been shown to be increased in the CSF of TBI patients and was accompanied by a loss of integrity of the BBB. Interestingly, several patients in this study experiencing secondary insults such as hypoxemia or hypoperfusion had more pronounced levels of C5b-9 in their CSF [90].

Complement protein synthesis has also been demonstrated in the brain after TBI both experimentally and clinically, with postmortem analysis of human brain tissue revealing the upregulation of C1q, C3b, C3d, and C5b-9 in close association with neurons in patients with focal brain contusions [91]. Experimentally, TBI-induced C3 deposition has been demonstrated by immunohistochemistry after lateral fluid percussion TBI [92].

The deleterious role of C5b-9 after TBI has also been demonstrated experimentally in mice null for the C5b-9 regulator, CD59. CD59 is able to prevent the formation of C5b-9 and thus acts as an essential inhibitor of complement activation and protector from cell death [93]. Consistent with its role, deletion of CD59 led to worsened neurological outcomes and heightened neuronal cell death, demonstrating the key role of the complement pathway is the pathophysiology of TBI [94]. This detrimental property was corroborated in transgenic mice overexpressing the soluble complement inhibitor Crry (complement receptor, related protein y), which had reduced neurological impairment and improved BBB dysfunction following TBI compared to wild type controls [95]. Furthermore, the pathogenesis of complement activation after TBI has been demonstrated by dual inhibition of both the classical and alternative pathways by pretreatment of rats with a soluble complement receptor type 1 (sCR1) prior to experimental weight-drop TBI. This dual pharmacological inhibition resulted in a significant decrease in posttraumatic neutrophil infiltration, suggesting that complement activation is an essential mediator of the early neutrophil inflammatory response after TBI [96]. Similarly, experimental TBI using mice deficient for C3 or the downstream C5, or treatment of wild type mice with the C5a receptor agonist lessened neutrophil extravasation and resulted in smaller lesions [88]. When C3 was injected intracerebrally into C3 deficient mice, the extravasation of neutrophils to the lesion site was amplified, suggesting that that locally produced C3 is important in brain inflammation [88].

## 7. Chemokines Mediate Posttraumatic Neuroinflammation and Tissue Damage

With the ability to dictate directional migration of neutrophils and leukocytes, chemokines are considered essential mediators of posttraumatic neuroinflammation as they control immune cell trafficking from circulation to extravasation [55, 97]. Two main families of chemokines have been described: CXC and CC. The CXC cytokines, including CXCL2, CXCL1, CXCL3, and CXCL8, are predominantly chemoattractant for neutrophils [65], whilst the CC chemokines CCL-2 (MCP-1) and RANTES attract monocytes and lymphocytes [66]. Additionally, a third class of chemokines has been implicated in the pathogenesis of brain injury, the CX3C subfamily, with the only characterised member being fractalkine (CX_3_CL1). Fractalkine has the unique ability to attract both neutrophils and monocytes, as well as T cells [98].

Clinically, CXCL8 has been found to be acutely elevated in the CSF and extracellular fluid of patients with severe TBI and correlated with BBB dysfunction and NGF production [99, 100]. In paediatric TBI, elevation of CXCL8 strongly correlated with mortality [101]. Severe TBI patients also experienced a sustained elevation in levels of CCL-2 for 10 days after injury, though this was highest on days 1 and 2 [97]. Using cerebral microdialysis, several groups have recently demonstrated acutely elevated levels of CCL-3, CCL-4, and RANTES after severe TBI [100, 102]. A prolonged elevation of fractalkine in the CSF has also been observed in patients after TBI, with a strong correlation to BBB dysfunction and corresponding low fractalkine levels in the serum [103].

Evidence suggests that CXCL1, and particularly CXCL2, are the key mediators of neutrophil migration early after focal brain injury, with both CXCL1 and CXCL2 found to be acutely upregulated within 5 h of experimental cortical impact injury in both mice and rats [61, 104], while after lateral fluid percussion injury CXCL2 expression has been shown to peak at 4 h in the injured hemisphere [105]. Using mice null for the CXCL2 receptor (CXCR2) in a cortical impact model, our group demonstrated a significant attenuation in the numbers of neutrophils migrating to the site of injury as early as 12 h after injury, and found that this correlated with reduced amounts of cell death and tissue damage [62].

Ample experimental evidence also exists to demonstrate the presence of monocyte-attracting chemokines acutely after injury, with elevated mRNA for CCL-2, CCL-4 and RANTES all observed after experimental cortical injury [97, 106]. By 4 h, production of CCL-2 and CCL-4 is significantly upregulated both *in vivo* and *in vitro* [61, 104, 107], with levels of CCL-2 peaking between 8 and 12 h after injury [97, 105]. Elevation of these chemokines after both focal and diffuse TBI is strongly correlated with poor functional outcome [46, 97], with more evidence of this provided using a CCL-2 knockout mouse for cortical injury, in which improved neurological function and reduced lesion volume were attributed to a reduction in macrophage accumulation [97].

This experimental evidence certainly suggests that chemokines play a deleterious role in the pathogenesis of focal brain injury, however their effects in diffuse brain injury are rather different, particularly with respect to CXC (neutrophil-attracting) chemokines. Without the presence of a gross pathological lesion, very low levels of CXCL2 have been observed in diffuse TBI, correlating with absent neutrophil migration into the brain [46]. However, diffuse TBI is associated with abundant accumulation of monocytes/activated microglia in the white matter tracts, colocalising with axonal pathology [28, 108, 109]. This cellular infiltration/activation also correlates with elevated CCL-2 levels acutely after diffuse injury [46]. So, it appears that CC chemokines play a more significant role in diffuse injury, whilst focal injuries involve both CXC and CC chemokines. These distinct molecular profiles very much reflect individual modes of cellular infiltration in these injury subsets.

## 8. Proinflammatory Cytokines Have Dual Roles in Traumatic Brain Injury

Proinflammatory cytokines are produced by several types of resident CNS cells such as microglia, astrocytes, and neurons in response to pathological challenge. Cytokines are usually preformed peptides that are activated by cleavage, and swiftly released in response to various stimuli. Once released, cytokines upregulate the expression of cell adhesion molecules and signal the secretion of chemokines in the early postinjury period [47], thus stimulating the infiltration of inflammatory cells to the injured regions. The activation of proinflammatory cytokines in human and rodent TBI has been reported since the early 1990s [99, 101, 102, 110–115]. Their role within the injured brain is, however, one of duality, in that they inherently promote repair, but often bring about additional tissue degeneration by activating a number of cytotoxic pathways leading to cell death [67]. It appears that both the timing of proinflammatory cytokine release and their concentrations are critical to ongoing secondary damage after TBI. The cytokines interleukin IL-1*β*, TNF, IL-6 and granulocyte-colony macrophage stimulating factor (GM-CSF) have been intensely investigated in a multitude of human and experimental paradigms to elucidate their role within the injured brain (see Table 1). Each of these cytokines is discussed in more detail below.

## 9. IL-1

IL-1 is known to induce many signaling pathways stimulating the production of other proinflammatory cytokines and thus is thought to be a key player in initiating the “cytokine cycle” [136]. IL-1 exists in both membrane-bound (IL-1*α*) and -secreted (IL-1*β*) forms, however it is IL-1*β* that has earned a reputation as the perpetrator of the acute inflammatory response to TBI. An important distinction is to be made, however, between IL-1*β* and other cytokines, in that IL-1*β* itself is not directly toxic when produced; rather it is the propensity to incite other cytokines that lends to its cytotoxic reputation. In noninjured tissue, IL-1*β* administration alone has been demonstrated to have no ill effects [137], however after TBI IL-1*β* mRNA is upregulated within minutes, and increased protein levels are detectable within an hour [110, 138–141]. Clinically, acutely elevated levels of IL-1*β* have been detected after injury by microdialysis [100, 102, 117], in patient CSF [116, 128], and directly in perioperative and postmortem brain tissue after TBI at both protein and mRNA levels [131, 142]. IL-1*β* levels have also been demonstrated to decrease rapidly; in rat models of focal cortical impact and lateral fluid percussion, IL-1*β* peaks at 6 h post-injury and returns to baseline by 72 h [143, 144]. This early and transient rise in IL-1*β* was also consistent with our recent findings in diffuse TBI, with a peak in IL-1*β* levels at 2 h in the cortex of rats subjected to diffuse TBI [27]. When combined with posttraumatic hypoxia, production of IL-1*β* was prolonged to 24 h, suggesting that this combinatory insult significantly amplified and sustained this early inflammatory response.

Evidence for the detrimental role of IL-1*β* is found in experiments in which its expression is modified, with neutralisation of IL-1*β* in a model of focal TBI in mice resulting in reduced tissue loss and improved visuospatial learning [145]. Furthermore, mice null for the IL-1 receptor (IL-1R1) had decreased VCAM-1 mRNA and a subsequently reduced extravasation of peripheral macrophages after stab wound injury. An overall reduction of inflammation resulted in fewer activated microglia and delayed and depressed expression of cerebral IL-1 and IL-6 [146]. Similarly, blockage of IL-1*β* signaling by use of an IL-1 receptor agonist (IL-1ra) has also been shown to delay the production of other proinflammatory cytokines, reduce cell death, and improve neurological recovery after experimental focal TBI and ischaemia [147, 148]. Clinically, endogenous IL-1ra microdialysate levels in have also been correlated with improved outcomes in TBI patients [117]. This largely negative role of IL-1*β* after injury has also been corroborated by peripheral administration of IL-1*β* after TBI, leading to larger lesions and impaired behavioural outcomes in rats subjected to fluid percussion injury [118].

## 10. TNF

Along with IL-1*β*, TNF has long been thought of as a cytokine of detriment following injury and still remains a subject of controversy, particularly as both cytokines have many signaling cascades in common and share the same physiologic effects, with the neurotoxic effects of IL-1*β* synergistically enhanced in the presence of TNF [149]. TNF is produced by microglia and astrocytes and its expression is regulated in an autocrine manner [150]. In TBI patients, high levels of TNF in the CSF have been observed acutely after injury [102, 120, 121], although the concentrations of TNF have been detected at considerably lower levels compared to other cytokines such as IL-6, TGF-*β*, and IL-8. TNF is also upregulated acutely in various experimental rat models of focal injury [115, 151] and has been fingered as a key mediator of the inflammatory response, with exogenous TNF administration in healthy brains causing breakdown down of the BBB and increasing recruitment of peripheral leukocytes [119, 123, 124]. Consistent with the hypothesised early detrimental role of TNF in the setting of TBI, its inhibition resulted in ameliorated BBB dysfunction [125] and decreased neuronal damage [152]. Whilst most of the evidence to date has documented the deleterious role of TNF in brain injury, this is increasingly becoming an issue of contention, particularly with longer-term studies of TNF-deficient mice, which showed a robust improvement in neurological function initially after TBI, but which then failed to progress in the long term compared to wild type mice [126]. In addition, TNF-deficient mice have also been shown to have exacerbated tissue and BBB damage after TBI [127]. These findings suggest a key detrimental role for TNF in the acute phase, but demonstrate that it may also have a crucial reparative role essential for long-term recovery. The intrigue of TNF action is not only of its temporal benefit or detriment, but also in its differential expression in focal and diffuse brain injuries and species-specific expression. Interestingly, the majority of studies examining TNF expression have used rat focal TBI models, and we and other groups have not observed any changes in TNF levels in rats subjected to diffuse TBI [27, 140], despite the fact that, like focal injuries, diffuse TBI evokes a substantial microglial and astrocytic response. However, when rats were subjected to diffuse TBI with posttraumatic hypoxia, our group showed a significant increase in TNF levels at 2 h, which was maintained until 72 h after injury [27]. In contrast to rat models of focal TBI, in the mouse closed head injury model we have not observed significant upregulation of TNF at any time examined [61, 97], and it is becoming increasingly apparent that there may be a species-specific production of TNF in CNS pathologies, in that rats produce more and mice less when subjected to similar levels of brain damage [153].

## 11. IL-6

IL-6 is a true pleiotropic cytokine, with roles in both pro- and anti-inflammation, and deleterious and beneficial effects after TBI [154–156]. However, it is known most often for its role as an immune stimulator, able to regulate chemokine production, cell adhesion molecule expression, and enhance leukocyte recruitment [157]. Clinical studies have indicated that IL-6 is, for the most part, neuroprotective, with maximal expression observed two days after injury [102, 112, 116] and CSF levels correlating with improved outcome in both children and adults [128, 129]. Previously, we have demonstrated an increase in IL-6 in the CSF over the first 24 h after mild experimental diffuse TBI, with production of both IL-6 mRNA and protein localised to neurons [130]. The most telling evidence of the beneficial role for IL-6 has come from studies of IL-6 gene deficient mice, which have been shown to have poor behavioural recovery, as well as increased oxidative stress, a more compromised immune response, and heightened neurodegeneration [158–160].

## 12. GM-CSF

Granulocyte-macrophage colony-stimulating factor (GM-CSF) is a hematopoietic cytokine produced by monocytes, macrophages, and endothelial cells [161], with its receptor expressed on most cell types in the CNS [162]. GM-CSF has been shown to have a positive role in promoting neuronal differentiation of adult stem cells *in vitro *[132], though as one of the least-examined cytokines after TBI, the role of GM-CSF is still largely to be elucidated. However, GM-CSF concentrations have been found to be significantly upregulated in human postmortem brain tissue within minutes of injury [131], indicating that GM-CSF plays an important role in the acute inflammatory response. This role appears to be one of neuroprotection, with a recent study employing stab-wound injury in rats observing that tissue loss was reduced by 40% when rats were administered a combination of exogenous GM-CSF and IL-3 [133]. Similarly, in models of rat spinal cord injury, rats treated with GM-CSF had reduced numbers of apoptotic cells and significantly improved neurological function [134] as well as reduced glial scar formation, preserved axonal cytoskeleton integrity, and higher numbers of regenerating axons [135]. In addition, rats exposed to focal cerebral ischemia had smaller infarct volumes and altered expression of apoptosis-related genes, with significantly increased levels of the antiapoptotic Bcl-2 and decreased levels of the pro-apoptotic genes Bax and p53 after treatment with GM-CSF [163]. In a mouse model of cerebral ischemia, GM-CSF administration also reduced the infarct size and increased the numbers of circulating blood monocytes/macrophages [164]. Taken together, these studies indicate that GM-CSF may play a beneficial role in neuroprotection, however more studies are required to clarify its full potential after TBI.

## 13. Toll-Like Receptors Mediate Innate Immune Responses to CNS Trauma

The toll-like receptors (TLRs) are a family of pattern recognition receptors which mediate innate immune responses to diverse pathogen-associated molecular patterns (PAMPs) [165]. Following injury or neurodegenerative disease without an infectious etiology, the engagement of danger-associated molecular patterns (DAMPs) by TLRs leads to exacerbated immune activation and enhanced neuropathology [166, 167]. Like all innate immune responses discussed here, TLR signaling is typically beneficial, yet it has become increasingly clear that following injury signaling through TLRs has particularly pathological consequences, contributing to the activation of microglia and subsequent induction of NF*κ*B leading to the transcription of proinflammatory mediators [168, 169]. Microglia are known to express all recognised TLRs [169], however the expression of TLRs on astrocytes is a contentious topic, with some researchers observing the presence of TLR-2 and TLR-4 mRNA in astrocyte culture [170], whilst others were unable to identify the expression of any TLR in 99% pure human astrocytic culture [171].

Many molecules may act as endogenous ligands for TLR signaling, with evidence suggesting that the TLRs involved most in TBI are TLR-2 and TLR-4, and that signaling through these TLRs triggers NF*κ*B activation and gene transcription [12]. Whilst research on the role of TLRs after TBI is scant, levels of TLR2 has been noted to be significantly upregulated after mouse bilateral cortical contusion [172], and significant infiltration of TLR-2 positive macrophages/microglia has been observed in the lesioned area and subcortical white matter after weight-drop injury in rats [173]. It appears though that the most compelling evidence of the roles of TLRs in TBI comes from experiments in which they are suppressed or deleted, with TLR-2 knockout mice showing an 18-fold reduction in GFAP mRNA, and 4-fold reduction in CD11b mRNA after stab-wound injury when compared to wild type. The authors also found less infiltrating astrocytes in the lesioned area, with those present possessing a less-activated morphology [174], suggesting that activation of TLR-2 was a substantial contributing factor to glial activation. In another study, suppression of TLR-4 using the monosomic alkaloid oxymatrine after focal TBI led to reduced gene expression of NF*κ*B and lower concentrations of TNF-*α*, IL-1*β*, and IL-6, with fewer apoptotic neurons as a consequence, suggesting a negative role for TLR-4 in neuroinflammation [175]. A double-knockout of TLR-2 and TLR-4 also resulted in decreased IL-1*β* and MCP-1 signaling after sciatic nerve damage, as well as significantly decreased macrophage recruitment/microglial activation, however these rats were noted to have poor locomotor recovery, impaired Wallerian degeneration, and inhibited axonal regeneration [176]. Interestingly, a single microinjection of the TLR-2 and TLR-4 ligands at the lesion site resulted in faster clearance of degenerating myelin, and significant and sustained improvement in motor function, indicating that while TLR signaling may be detrimental in terms of the acute neuroinflammatory response, it may in fact be important for long term recovery in terms of myelin clearance and nerve regeneration [176].

## 14. Immunotherapies for TBI

Despite more than 30 years of research, not a single effective therapy has been developed for the treatment of TBI. A multitude of compounds showing promise in animal studies have failed to exhibit beneficial effects in clinical trials, with more than 20 compounds reaching phase II/III trials but showing no long-term benefit [7]. In one of the largest clinical trials for TBI to date, the corticosteroid randomisation after significant head injury (CRASH) trial investigators found that despite encouraging results in animal studies in which corticosteroid treatment was found to be efficacious, in a clinical setting the administration of corticosteroids after TBI was strongly correlated with excess mortality [177].

The lack of success of clinical trials has been attributed to several factors, including superficial examination in animal models with premature translation to the clinic, variations in therapeutic windows in animals and humans and variable dosing schedules, and failure of experimental models to include secondary insults which are commonplace in clinical TBI. Finally, animal models of TBI are by design well-controlled and reproducible, whilst clinical TBIs are far more complex and inherently heterogeneous [178, 179]. In order to address these problems, experimental studies are increasingly employing more clinically relevant species with secondary insults, and many compounds are trialed in larger animal models in order to establish efficacy in more clinically relevant brains before moving to clinical trial. Compounds that are currently under investigation for the treatment of TBI fall broadly into two categories: those with multiple targets and modes of action in CNS pathologies, and those with a single target of action. Examples of each with relevance to innate immunity are presented below.

### 14.1. Compounds of Multifunctional Modality

#### 14.1.1. Erythropoietin

Erythropoietin (Epo) is a haematopoietic cytokine produced mainly by the kidney which is rapidly upregulated in response to hypoxia [180]. Epo has been used extensively in the treatment of chronic renal and anaemic patients and has been shown to reduce mortality in trauma patients [181]. In recent years, Epo has been highlighted as a promising neuroprotective candidate due to its current clinical use with few side effects and feasible therapeutic window of ~6 hours [182]. Epo and its receptor EpoR are rapidly upregulated in the brain after various insult models [183], and its administration after experimental injury was shown to be efficacious in a number of experimental TBI paradigms. Importantly for the treatment of TBI, Epo has numerous targets in the brain, with robust benefits including anti-inflammation, with a reduction in immune mediators' levels and subsequent reduction in inflammatory cell infiltrates, diminished cell death, reduction of oedema, rectification of BBB dysfunction, resolution of cerebral vasospasm, as well as enhanced neurogenesis, and angiogenesis and improvement in sensorimotor function [183–187]. Currently, Epo is being investigated in a phase III clinical trial within multiple sites in Australia, with an estimated completion date of 2014.

#### 14.1.2. Minocycline

The tetracycline derivative minocycline has been posited as a neuroprotective candidate in several experimental models of CNS injury due to its potent anti-inflammatory actions [61]. After focal TBI in mice, minocycline has been shown to attenuate microglial activation and reduce the expression of IL-1*β* [61, 188, 189], as well as acutely reduce the size of focal brain lesions [61, 188, 190] and decrease cerebral oedema [189]. Minocycline may also improve neurological function, however several studies report this effect may be transient, with beneficial outcomes only observed acutely [61, 190]. Minocycline is currently being investigated in a phase I trial in Detroit, Michigan, with imminent completion.

#### 14.1.3. Progesterone

The hormone progesterone has been shown to have multiple functions in the treatment of brain injury, and is able to exert its effects through steroidal, neuroactive and neurosteroidal mechanisms [191]. Experimentally, progesterone acts as a potent anti-inflammatory agent by dampening the cytokine response and limiting immune cell activation and extravasation [192], as well as decreasing NF*κ*B-mediated inflammatory gene transcription [193]. Progesterone has proved to be particularly effective in the treatment of focal brain injuries, in which it has been demonstrated to reduce neuronal damage, minimise oedema and improve neurological outcomes in a variety of focal contusion models [194–198]. Progesterone can also affect the complement system, with significant reductions in C3 cleaved fragments observed after bilateral frontal contusion in rats [193]. Although limited in number, studies of progesterone's effects on diffuse TBI have also demonstrated benefit with a reported reduction in BBB permeability and subsequent oedema [199] as well as a decreased number of apoptotic cells and the apoptotic precursor caspase 3, and a substantial decline in axonal pathology [200]. Importantly for translation to the clinic, delaying the administration of progesterone for 24 h still resulted in benefit, with a diminished oedema observed after cortical contusion injury [201]. Due to these benefits in rodent models, progesterone has been applied clinically, with evidence from the ProTECT clinical trial and other pilot studies suggesting that progesterone may reduce mortality and improve neurological outcomes after TBI [202, 203], warranting further investigation in a large multicentre trial. The ProTECT trial has now entered phase III, with an estimated completion date of 2015.

### 14.2. Single-Target Compounds

#### 14.2.1. Complement Inhibition

The complement system presents as an attractive target for immune modulation after TBI due to its prominent role in inflammatory cell extravasation. Several aspects of the complement system are amenable to interventions such as selective antagonists, making them viable candidates for clinical translation. Experimentally, administration of the soluble complement receptor 1 after weight drop injury in the rat significantly attenuates neutrophil infiltration into the injured brain [96]. A similar effect on neutrophil extravasation was also observed after cryoinjury in mice with deletion of either the C3 or C5 gene, or administration of the C5a receptor antagonist, with corresponding reductions in the chemokines CCL5 and CCL2, and smaller lesions as a consequence [88]. Inhibition of the alternative pathway has also shown promising results, with targeted deletion of the factor B gene or delivery of antifactor B neutralising antibody resulting in significantly decreased C5a serum levels and a reduction in cell apoptosis [204, 205]. Although it appears that targeting the complement system in the acute phase may be beneficial, it may also have deleterious consequences for long-term recovery. For example, treatment with the C5a receptor antagonist in rats after spinal cord injury resulted in a significantly less macrophages/microglia in the injury site at 7 days, however these rats also had poor locomotor recovery and reduced myelination, suggesting that while early inhibition of C5a may be beneficial, the long-term outcome of reducing this aspect of inflammation is detrimental [206].

#### 14.2.2. Anticytokine Antibodies

Whilst cytokines appear a natural target for neutralisation as the perpetuators of the inflammatory response, they must be considered in the context of the whole organism, in that the beneficial effects of abolishing such a targeted response may have more broad adverse consequences in recovery. Studies suggest, however, that there may be some benefit to inhibiting the actions of several cytokines, with neutralisation of IL-1*β* after focal TBI in mice attenuating neutrophil infiltration and microglial activation, minimising the number of ICAM-1 positive cells, and reducing oedema and improving cognitive outcome [145, 207, 208]. Treatment of mice with the IL-1ra has also resulted in benefit in various models and species, with better behavioural scores and attenuation of oxidative stress, as well as smaller lesion volumes [209, 210]. Importantly for the clinic, IL-1ra is able to penetrate the BBB in concentrations considered to be experimentally therapeutic [211], and even when administration is delayed by 4 h under experimental conditions, smaller lesion volumes are still observed in an animal model of TBI [210].

Therapeutic inhibition of TNF has also been demonstrated with good result after closed head injury in rats, with a reduction in oedema and recovery of motor function reported [125, 212]. However, this effect may vary depending on the model of TBI employed, with other researchers finding no benefit when employing neutralising antibodies to TNF in a lateral fluid percussion injury in the rat with respect to oedema, motor, or cognitive outcomes up to one week after injury [213]. Results of TNF neutralisation may also vary between species, with no effect observed on behavioural outcomes, lesion volumes or cell death in mice subjected to closed head injury [214].

#### 14.2.3. Antibodies to Cell Adhesion Molecules

Targeting the passage of immune cells through the BBB via inhibition of cell adhesion molecules presents an interesting avenue to dampen the neuroinflammatory response to TBI. Experimentally, administration of antibodies to ICAM-1 resulted in a substantial decrease in neutrophil recruitment [56, 122], however neutrophil accumulation was not completely abolished, thus suggesting a prominent role for other cell adhesion molecules in the absence of ICAM-1 [56]. Neutralisation of ICAM-1 also significantly improved motor performance after lateral fluid percussion injury in the rat, however a significant effect was also seen with IgG injection, indicating that there may be a nonspecific antibody effect [122]. In mice deficient in ICAM-1, however, no beneficial effect was observed with regard to neutrophil accumulation, lesion volume, or motor or cognitive function [215]. In mice double knockout mice for both ICAM-1 and P-selectin, whilst a significant reduction in oedema was observed, no differences to wild type were found with regard to histopathology, motor or cognitive function [216], providing more supporting evidence for a compensatory role of other cell adhesion molecules.

## 15. Summary

The innate immune response plays an intrinsic role in the governance of TBI, with both beneficial and deleterious consequences. This response is largely mediated by resident innate immune cells (microglia and astrocytes), while passage of peripheral immune cells into the brain is facilitated by opening of the BBB, or by upregulation of adhesion molecules and chemokines to aid their movement into the injured tissue. Chemokines such as CXCL2 and CCL-2, and cytokines such as IL-1*β*, TNF, and IL-6 also play essential roles in dictating migration and recruitment of immune cells to sites of injury, with reparative or destructive consequences depending on the timing of their release and their concentrations. Whilst the intention of the innate immune response is to promote repair, restorative efforts are often hampered by the presence of additional inflammatory factors such as complement proteins and increased signaling through microglial TLRs, which results in a disproportionate and self-perpetuating immune response. This dysregulation has become a key target for therapeutic intervention, with both single-target and multifunctional drugs evaluated in efforts to curb the innate immune response. Therapeutic targets are wide ranging, with a focus ranging from adhesion molecules to cytokines in an effort to minimise cell entry, activation and expansion. As yet, no one compound has proven efficacious when applied in multiple models or translated to the clinic, highlighting the need for more rigorous investigation in multiple pathological scenarios prior to clinical application.

## 16. Conclusion

It has become increasingly clear over the last two decades that the innate immune system plays a crucial role in the pathogenesis of TBI. The innate immune system is, by nature, complex and interrelated, with each crucial aspect shaping the structure for the next, and ultimately determining the outcome following TBI. It is this intricate nature, however, which heightens the challenge faced by researchers and clinicians alike in both understanding and combating the secondary consequences of brain trauma. While research into the pathogenesis of TBI is rapidly advancing, many of the complex interactions between compartments of the innate immune response are still unknown. However, with further understanding and more thorough preclinical screening of neuroprotective candidates, the development of an effective therapy for the treatment of TBI could be achieved.

## Figures and Tables

**Table 1a:** (a)

IL-1*β*			
Finding	Clinical/experimental	Experimental setting	Reference

Acutely upregulated after TBI	Clinical	Cerebral microdialysis; adult and pediatric patient CSF	[100, 102, 116, 117]
Peripheral administration after TBI results in larger lesions and impaired behavioural outcomes	Experimental (rat)	Fluid percussion injury	[118]
Expression exacerbated and prolonged by secondary insult	Experimental (rat)	Diffuse axonal injury with posttraumatic hypoxia	[27]
Causes BBB dysfunction *in vivo *	Experimental (rat; *in vitro*)	Cerebral endothelial cells	[119]

**Table 1b:** (b)

TNF			
Finding	Clinical/experimental	Study methodology	Reference

High levels observed acutely after injury	Clinical	Cerebral microdialysis, adult patient CSF	N [102, 120, 121]
Acutely upregulated in rats after focal TBI	Experimental (rat)	Controlled cortical injury; lateral fluid percussion	[115, 122]
Administration causes BBB dysfunction and increased recruitment of peripheral leukocytes	Experimental (rat, newborn piglet, rat; *in vitro*)	Healthy animals/cerebral endothelial cells	N [119, 123, 124]
Inhibition of TNF ameliorates BBB dysfunction	Experimental (rat)	Controlled cortical injury	[125]
Deficiency of TNF/TNF-R causes exacerbated BBB damage and impairs long-term recovery	Experimental (mouse)	Controlled cortical injury	N [126, 127]
Expression exacerbated and prolonged by secondary insult	Experimental (rat)	Diffuse axonal injury with posttraumatic hypoxia	[27]

**Table 1c:** (c)

IL-6			
Finding	Clinical/experimental	Study methodology	Reference

CSF levels correlate with improved outcome	Clinical	Adult and pediatric patient CSF	[128, 129]
Production within 24 h localised to neurons	Experimental (rat)	Diffuse axonal injury	[130]
IL-6 deficient mice have heightened neurodegeneration, increased oxidative stress, poor behavioural recovery	Experimental (mouse)	Controlled cortical injury; aseptic cerebral injury	[158–160]

**Table 1d:** (d)

GM-CSF			
Finding	Clinical/experimental	Study methodology	Reference

Significantly upregulated in brain tissue within minutes of TBI	Clinical	Postmortem brain tissue	[131]
Promotes neuronal stem cell differentiation *in vitro *	Experimental (rat; *in vitro*)	Neural stem cell culture	[132]
Promotes tissue sparing when administered in conjunction with IL-3	Experimental (rat)	Stab-wound injury	[133]
Minimises tissue damage and promotes behavioural recovery	Experimental (rat)	Spinal cord contusion	[134, 135]
